# Construction of membrane-bound artificial cells using microfluidics: a new frontier in bottom-up synthetic biology

**DOI:** 10.1042/BST20160052

**Published:** 2016-06-09

**Authors:** Yuval Elani

**Affiliations:** * Department of Chemistry, Imperial College London, Exhibition Road, London SW7 2AZ, U.K.

**Keywords:** artificial cells, biomimetics, droplet interface bilayers, microfluidics, synthetic biology, vesicles

## Abstract

The quest to construct artificial cells from the bottom-up using simple building blocks has received much attention over recent decades and is one of the grand challenges in synthetic biology. Cell mimics that are encapsulated by lipid membranes are a particularly powerful class of artificial cells due to their biocompatibility and the ability to reconstitute biological machinery within them. One of the key obstacles in the field centres on the following: how can membrane-based artificial cells be generated in a controlled way and in high-throughput? In particular, how can they be constructed to have precisely defined parameters including size, biomolecular composition and spatial organization? Microfluidic generation strategies have proved instrumental in addressing these questions. This article will outline some of the major principles underpinning membrane-based artificial cells and their construction using microfluidics, and will detail some recent landmarks that have been achieved.

## Introduction

Synthetic biology concerns itself with the construction of biological systems distinct from those seen in nature. The discipline can be tackled from two opposing directions. The first and better developed approach is to take living cells and to alter them so that new functions arise (the top-down approach). In recent years an alternative strategy has emerged: starting with simple chemical or biological components and building up artificial biomimetic structures from scratch (the bottom-up approach) [1–3]. These synthetic structures resemble biological cells in form and/or function, and are widely known as artificial cells, although alternative terminology includes synthetic cells, protocells, minimal cells and semi-synthetic cells. They can contain biological building blocks and cellular machinery (lipids, enzymes, membrane channels, structural proteins), and can possess their own rudimentary synthetic genomes. They can also contain synthetic elements (polymers, nanoparticles, electronic interfaces).

The boundaries of artificial cells need to be defined by a barrier delineating their interior from exterior, analogous to plasma membranes in biological cells. This barrier can be based on polymers [4], coacervates [5–6], water droplets [7], pickering emulsions [8] and proteinosomes [9]. One of the most attractive classes of artificial cells are those based on lipid membranes, as their biocompatibility and similarity to biological membranes enables cellular machinery to be reconstituted without loss of function. Lipid membrane based artificial cells generated from the bottom-up are the subject of this article, with a particular focus on how microfluidic technologies are revolutionizing the way in which they are generated.

Artificial cells have been envisaged as the basis of a new generation of biomimetic soft-matter devices that are capable, for example of, self-repair, of responding to their environment and of communicating with biological cells. Prospective applications for such cells are diverse, ranging from on-site responsive drug synthesis and targeted delivery [10], to *in vivo* diagnostics and programmable microreactors [11]. Artificial cells can also be used as models for biological cells, enabling biological systems to be studied in a simplified and controlled environment.

Building cells from the bottom-up, as opposed to simply modifying existing cells, has several inherent advantages. Non-biological building blocks which would ordinarily interfere with cellular processes can be incorporated. Molecules and intermediates that would be toxic to biological cells can be produced. As artificial cells can be engineered to perform specific, singular functions, resources and energy do not need to be wasted on the multitude of auxiliary functions that biological cells perform. The complexity of artificial cells is much reduced, meaning that full control over variables can be maintained, making artificial cells easier to study, design and control. Finally, the fact that artificial cells are not living makes them attractive from an ethical, safety and public perception standpoint.

Research into the construction of artificial cells has experienced a surge in recent years. One of the main drivers behind this has been the emergence of microfluidics as an enabling technology for their generation, manipulation and analysis. The question then arises: what is it about microfluidics that makes it so attractive to bottom-up synthetic biology? Why are these fields so synergistic? By exploring the principles underpinning the discipline of artificial cells, by examining the basic concepts behind microfluidics and by detailing recent case studies, these questions are addressed herein.

## Membrane-bound artificial cells

**Figure 1:**
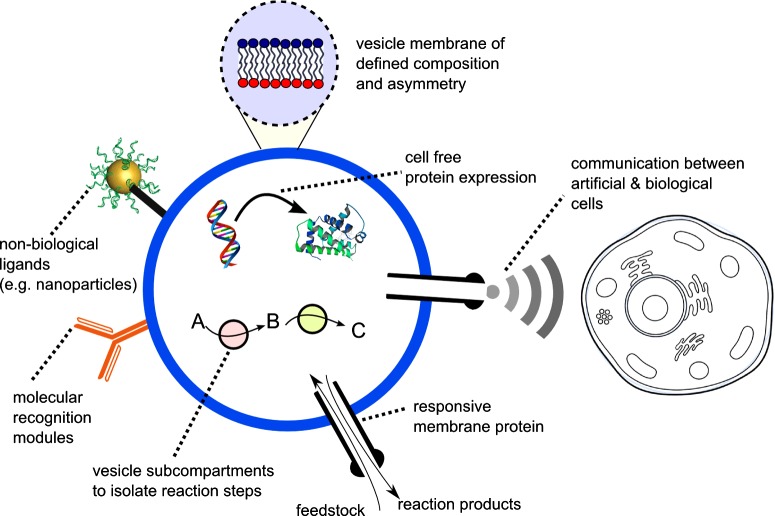
Schematic of a hypothetical vesicle-based artificial cell which contains some key cellular components and features (i) Membrane of defined biomolecular composition and asymmetry. (ii) Dynamic cell-free expression of proteins by IVTT using rudimentary genetic circuits. (iii) Incorporation of non-biological components. (iv) Communication between an artificial cell and a biological cell via an engineered signalling cascade. (v) Embedded responsive protein pores that open/close according to external stimuli. (vi) Membrane-embedded recognition modules (e.g. antibodies). (vii) Sub-compartmentalization inside cells into regions with distinct chemical environments for multi-step reactions.

Artificial cells can have a range of synthetic and biological modules incorporated within them, giving them functionality (Figure 1). Typically, the surrounding membrane take the form of lipid vesicles, which vary in diameter from 100 nm to 100 μm, and are thus in cellular size regimes. The vesicle membranes encapsulate material and allow concentration gradients to be generated. Furthermore, by reconstituting appropriate biological machinery into membranes key cellular processes can be recapitulated, including the uptake of nutrients and expulsion of waste [12], intra-cellular signalling cascades [13], communication with other cells [14–15], replication and division [16–17] and limited evolution [18].

Vesicles can be loaded with a variety of chemical cargos and biomolecules, including DNA, enzymes and small molecules. They can contain purified cell lysates (either commercially bought or developed in-house), which enables cell-free expression of defined proteins via *in vitro* transcription and translation (IVTT). Artificial cells that are capable of generating their own cytoskeleton [4], of synthesizing enzymes and membrane protein pores [12], of amplifying DNA [17] and of dynamic protein expression using genetic circuits can now routinely be generated [19].

Crucially, as one of the aims of bottom-up synthetic biology is to create designer cells with properties that can be precisely defined, the features of the membrane and encapsulated materials need to be controlled. The most important variables associated with artificial cells include: (i) their absolute size, (ii) their size distribution (i.e. how homogeneous the population is), (iii) biomolecular content and the lateral organization of the membrane, (iv) biomolecular content of the interior and (v) sub-compartmentalization and spatial organization of encapsulated material.

Control of these variables are especially important if artificial cells are to be tailored for applications such as drug delivery, as tissue mimics, as simplified models to investigate biological phenomena, for drug screens or as soft and smart devices. It is due to this fine control of vesicle parameters, coupled with the capability for high-throughput and on-demand generation that microfluidics has a significant role to play.

## Microfluidics

Microfluidic systems involve fluids that are confined in the micrometre size regime (1–1000 μm). They are often contained ‘on-chip’, using devices which are connected to pumps which drive flow. These are analogous to microelectronic chips (indeed, fabrication methods have been borrowed from the electronic industry), but instead of processing electrical information around a circuit, microfluidic chips are used to manipulate fluid chemical and biological materials.

The physics of fluids differ when confined in microfluidic channels [20] with viscous forces dominating over inertial forces, a relationship given by the Reynolds number (*R_e_*), which is dependent on the fluid density (*ρ*), flow rate (*v*), hydraulic diameter (*D*_h_) and viscosity (*μ*):

$$\;R_{e}=\frac{\rho vD_{\;h}}{\mu }\;$$

Due to small channel dimensions, microfluidic systems typically have Reynolds numbers smaller than 2300, with laminar flow dominating over turbid flow.

The advantages of microfluidic technologies are many and varied. Sample sizes are low, which reduces cost and allows scarce materials to be used. Miniaturization enhances portability and enables parallelization. Microfluidic devices are often designed to be plug-and-play, generally have enhanced resolution and performance, and lend themselves to high-throughput applications, particularly with the emergence of droplet-based microfluidics [21].

## Droplet microfluidics for vesicle construction

Microfluidic devices of varying designs can generate droplets of defined volumes. [21] For biological applications these droplets tend to be water-in-oil, although oil-in-water droplets can also be generated. In top-down synthetic biology, droplet microfluidics has been used to encapsulate single cells and DNA strands in single droplets, for directed evolution purposes, for whole cell analysis and for gene expression profiling [22–23].

However when it comes to the construction of artificial cells from the bottom-up, droplet microfluidics has proved to be transformative. Recent developments have enabled droplets to be used as precursors to lipid vesicles by assembling a bilayer around the droplet exterior, with the content of the droplet becoming the interior of the vesicle-based cell [24]. This allows the encapsulation of large, charged molecules (proteins, enzymes, DNA) for the first time, with encapsulation efficiency approaching 100%. Biochemical processes can therefore be incorporated in the cell interior, a prerequisite for the construction of artificial cells.

The use of droplets also makes it possible to exploit the unique advantages inherent in droplet microfluidics (in addition to the more generic benefits of microfluidics as whole, mentioned above), including:

- The production of droplets of defined size, from 500 nm to 500 μm in diameter
- Uniform size distribution of droplets (approximately 3% coefficient of variation)
- High-throughput generation of droplets (2 kHz)
- On-site and on-demand droplet manufacture
- Multiplexing with other microfluidic modules to perform unit operations (merging, mixing, sorting, storing, concentration etc.) [21]

Over the last decade there have been a wide array of examples of the generation of vesicles and artificial cells using droplet microfluidics. Some key milestones are highlighted below, although the reader should be aware that this is not a comprehensive account, but one that indicates the scope of recent developments.

### Vesicle generation using phase transfer

**Figure 2:**
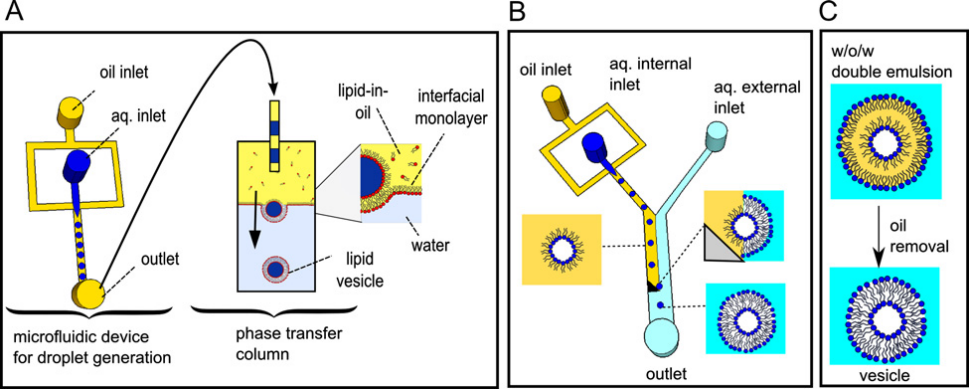
Strategies for microfluidic generation of vesicles (**A**) Droplets are formed on-chip, and then expelled above an oil–water column. Lipid is dissolved in the oil phase, and an interfacial monolayer assembles around the droplet and at the water/oil interface of the column. The droplets are loaded with sucrose, and therefore descend through the column under gravity. As droplets enter the lower water phase they are encased in a second interfacial monolayer, resulting in a bilayer membrane. (**B**) Schematic of a device where both droplet generation, and subsequent conversion into vesicles, occurs on chip, with the aid of a triangular microfabricated post, guiding droplets across the phase boundary. (**C**) Vesicle generation from double emulsions formed with a microfluidic device. The emulsions are stabilized by lipids and, as the intermediate oil phase is extracted into the external phase, vesicles are generated.

One of the most well-established methods to transform droplets into vesicles uses a process termed phase transfer (Figure 2A) [25–27]. First, monodisperse water-in-oil droplets are generated on-chip with lipid dissolved in the oil [26], leading to an interfacial lipid monolayer encasing the droplets. These droplets are then expelled into a two-phase column, with the lower phase containing water, and the upper phase containing a second lipid-in-oil solution. Again, the presence of lipid leads to an assembly of a monolayer between the two phases of the column. The lipid-coated droplets, which are made heavier than both phases in the column through the addition of sucrose, then descend through the column due to the density difference. As the droplets pass from the oil into the water phase a second interfacial monolayer wraps around them, transforming them into vesicles. It is important to note that although droplets are generated on-chip, their transformation into vesicles is achieved in bulk-scale, off-chip.

### Vesicle generation on-chip

Others have developed a two-module microfluidic device which could accomplish *both* droplet generation *and* phase transfer on a single microfluidic chip [28] (Figure 2B). Droplets were generated in the first module using a flow focusing junction, and in a second module they were transferred into an aqueous phase with the aid of a micro-fabricated post, converting them into vesicles. A variation of this method is to use a step junction and channel depth variations to achieve phase transfer [29]. When the bending rigidities of these vesicles were analysed their mechanics were revealed to be the same as vesicles formed via traditional non-microfluidic techniques, demonstrating the potential for the incorporation of mechanosensitive membrane proteins as responsive modules in artificial cells. A related technique involved applying a pulsed jet flow to a planar lipid bilayer, yielding vesicles in an analogous manner to blowing a soap bubble from a soap film [30].

### Generation of vesicles using double emulsions

Another class of vesicle-generation strategy involves water–oil–water droplets known as double emulsions [31,32] (Figure 2C). These are formed on a microfluidic chip using sequential droplet generation and encapsulation in a larger droplet. When the intermediate oil phase contains lipid dissolved in an oil which is partially soluble in water, vesicles are formed as the intermediate phase is depleted, leaving behind a lipid bilayer. Vesicles generated using such methods have been shown capable of incorporating bacterial divisome protein filaments (FtsZ and ZipA) in their inner leaflets [31], opening up the possibility of using these to produce dividing artificial cells.

### Control over membrane asymmetry and lamellarity

Membrane asymmetry refers to a difference in lipid composition between the inner and outer leaflets of the membrane. This is a universal feature of biological membranes and is thought to play a key role in a host of cellular processes, including endo- and exocytosis, in the folding and gating of membrane proteins, in regulating membrane mechanics and in the activation of mechanoresponsive proteins [27,33]. For artificial cells to reliably replicate biological cells and their membranes, this feature needs to be incorporated. In addition, as most membrane are unilamellar (i.e. with only a single bilayer as opposed to multiple shells in an onion-like arrangement), control over this is also key.

Recently, microfluidic devices capable of generating vesicles with defined asymmetry and lamellarity have been developed [34]. These devices trapped water droplets on a series of fabricated structures in a microfluidic chamber. Individual leaflets of the membrane were then deposited around the droplet by sequentially flushing the device with an oil/water interface that was separated by a lipid monolayer. Different lipids could be used when assembling the two leaflets resulting in asymmetric membranes, and this process could be repeated multiple times leading to membranes with multiple layers.

### Alternatives to vesicle-based cells

There have been several successes in generating non-vesicle artificial cells using microfluidics. These include coacervates (spherical aggregates of colloidal droplets) that were formed using flow focusing chip geometry [6]. These were shown to be capable of *in vitro* gene expression [5], and coacervate populations containing different DNA oligonucleotides were shown to coexist without exchange of genetic information, demonstrating their suitability as artificial cell models. There have also been examples of polymer-based vesicles (polymersomes) generated using capillary microfluidic devices, which were capable of protein synthesis and triggered release of contents [4].

## Compartmentalized artificial cells

Cells have a complex but well-defined spatial organization of content and function, with different processes occurring in distinct cellular regions. In eukaryotes this is achieved by sub-compartmentalization using organelles, which allows them to carry out multiple functions concurrently, each within a distinct chemical/biochemical environment. The benefits associated with compartmentalization also apply to artificial cells and there have been several examples of using microfluidics to generate vesicle-based cells with defined compartmentalization and spatial organization.

**Figure 3:**
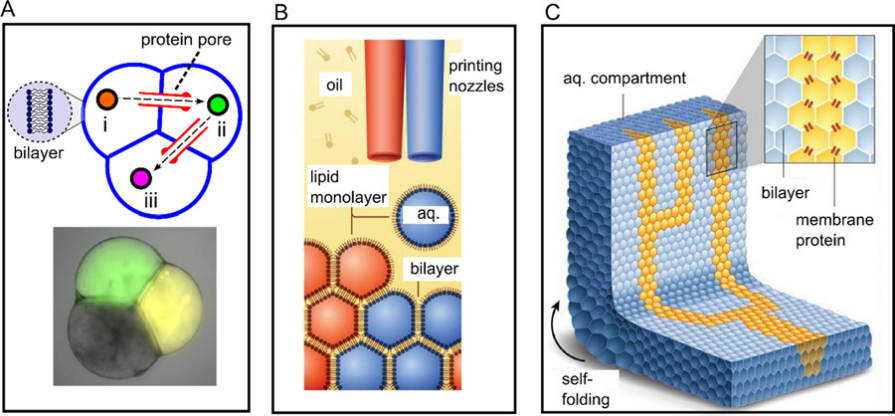
Higher-order membrane assemblies (**A**) Schematic of a three-compartment vesicle-based cell constructed from droplet precursors, where each compartment exists in a distinct biochemical environment, and is engineered to perform one step of a multi-step enzymatic reaction. The reaction intermediates move between compartments via transmembrane pores. (**B**) Schematic of a bespoke droplet printer capable of printing thousands of lipid-coated droplets, leading to a tissue-like material. (**C**) This artificial tissue can be functionalized to exhibit collective properties, including self-folding and transmittance of electrical signals down pre-defined paths through transmembrane proteins. Panels B and C were modified from [46]: Villar, G., Graham, A.D. and Bayley, H. (2013) A tissue-like printed material. Science **340**, 48–52, reprinted with permission from AAAS.

One such system is multi-compartment vesicles [13,35,36]. These are vesicles that have lipid bilayers spanning their internal volumes, thus partitioning them into distinct compartments (Figure 3A). These were generated using a variation of the phase transfer processes discussed previously, where multiple droplets were controllably transferred though an oil–water interface *together,* thus encasing them in a single bilayer. Recently, high-throughput microfluidic strategies for the generation of analogous structures have been developed, which also led to a reduction in their volumes to the pL regime [37].

The use of droplets means that all the key variables of these multi-compartment vesicles can be controlled [35]. The number and size of compartments can be precisely defined, with two-, three- or four-compartment structures reliably generated. Both the lipid composition and the content of each compartment can be controlled, meaning that compartments can carry distinct cargoes and be engineered to perform specific tasks. This was recently demonstrated by the *in vitro* synthesis of different proteins in each compartment, paving the way for the construction of multi-functional artificial cells, and allowing some of the complex spatial organization in biological cells to be recreated in artificial ones [36].

Importantly, such vesicles can be functionalized with transmembrane pores, allowing them to take up materials from their environment, and enabling communication between individual compartments via signalling cascades. A recent paper demonstrated the construction of a three-compartment vesicle with each compartment containing enzymes for one step of a multi-step reaction (Figure 3A) [13]. Each step in the enzymatic pathway could thus take place in an isolated chamber, with the reaction intermediates moving between compartments through embedded pores, finally yielding a fluorescent reaction product. This demonstrated the potential of vesicle-based artificial cells to act as pL reaction vessels in a physiological environment, which is significant for on-site *in vivo* drug synthesis applications.

There have also been examples of generating multi-compartment polymersomes on-chip in high-throughput, which were formed by generating double emulsion with two encapsulated internal droplets, followed by removal of the intermediate oil phase via oil-depletion methods [38].

## Artificial multicellular structures and larger-scale assemblies

As the field of artificial cells has advanced there have been several forays into exploring the concept of connecting several individual cells together to create multi-unit assemblies. These can be considered analogous to multicellular structures in biology (e.g. tissues), and in the same way, can exhibit collective and higher-order properties that cannot be engineered into single unit artificial cells.

One of the platforms used to achieve this is droplet interface bilayers (DIBs) [39]. If water-in-oil droplets coated with a lipid monolayer are brought into contact then a bilayer membrane is formed at the interface. By bringing together three or more droplets a network of DIBs can be generated. These networks of cell-like compartments separated by a membrane–whose architecture is defined simply by the location of the droplets–can be extended to contain tens, hundreds or thousands of droplets.

By appropriate functionalization with engineered transmembrane proteins, DIB networks that act as soft matter bio-devices have been constructed. These include simple electrical circuits, batteries for autonomous energy generation and light-harvesting devices and sensors based on the light-driven pump bacteriorhodopsin [39]. DIBs have been used as simplified models of cell membranes for applications such as rapid screening of channel blockers [39], as tools to better understand biological phenomena including the effects of membrane asymmetry on protein channels [41], and for probing the *in vitro* synthesis of membrane proteins and subsequent insertion into bilayers [42]. Recent advances have allowed DIB networks to be encapsulated in larger oil-in-water droplets to yield compartmentalized cell-like structures called multisomes [37,43]. This has opened up the exciting possibility of extending the power and functionality of DIB networks to aqueous (i.e. physiological) environments.

With DIBs being a droplet-based technique, microfluidics has proved a particularly valuable technology, primarily due to the ease and robustness with which droplets of cellular dimensions (approaching the micron scale) can be generated in high-throughput and subsequently manipulated. Microfluidics has enabled 2D and 3D DIB networks consisting of thousands of droplets to be generated, whose architectures were defined by the contours of a microfluidic chip [44]. More recently, the use of droplet-on-rail technologies has allowed more accurate positioning of droplets, which has enabled parallel DIB networks of defined sizes to be constructed on a single device [45]. Inter-droplet communication via an enzymatic reaction was shown in this work, demonstrating the potential of DIB networks to mimic signalling cascades. Finally, using sequential droplet generation and encapsulation, high-throughput production of cell-sized multisomes using double emulsion templates has been achieved, and the potential of these as programmable chemical reactors for the on-site synthesis of drugs demonstrated [37].

Villar et al. [46] used 3D printing of individual droplets to generate 3D DIB networks that resembled tissues. By functionalizing these with biomolecules they were able to transmit electric signals down defined paths in a manner analogous to neurons. The networks could further be engineered to exhibit cooperative behaviours, such as folding from a sheet into a hollow sphere, using osmotic differences between individual droplets in the networks to drive a change in geometry over time. Finally, they estimated the Young's modulus of the material to be similar to that of brain tissue and fat, demonstrating that extended DIB networks resemble biological tissues in this regard as well.

Although the discussion above has been limited to droplet networks, there is also an increasing interest in constructing vesicle clusters, either using complimentary DNA as tethers (enabling specific recognition between vesicles) or using avidin–biotin binding [47]. For now, however, the use of microfluidic platforms to assemble these has been limited.

The above examples demonstrate the extension of the concept of modularity that is central in synthetic biology. Traditionally, this refers to modular genetic parts (or bio-bricks), which are well characterized and can be joined together to create devices and systems with more complex properties in a predictable manner. In the context of bottom-up artificial cells however, the modules are droplets (or a collection of droplets) each with well-characterized features, serving distinct functions, that can be linked together in a network to yield higher-order features.

## Artificial cell-on-a-chip

Instead of employing microfluidic devices to produce vesicles as cell models, an alternative approach is to use the microfluidic chips *themselves* as artificial cells. This was elegantly demonstrated by Karzbrun et al. [48] who grafted a DNA brush on the microfluidic device substrate. A cell-free expression mixture was fed into the device, and fluorescent protein was continually produced. The use of a microfluidic device instead of a vesicle as a chassis allowed constant influx of building blocks into the channels and removal of reaction products. This effectively gave the proteins a lifetime, and, together with the use of appropriate genetic circuitry, allowed dynamic expression profiles to be observed including fluorescent oscillations driven by a negative feedback loop. Crucially, the behaviours seen could be altered simply by altering the device architecture (specifically the channel length), without changing the genome itself. Others have used similar microfluidic-based approaches to study protein–protein interaction networks and biochemical pathways [49], as well as the assembly of steady-state biological networks [50]. Such systems demonstrate the potential of cell-on-a-chip to be used to study biological networks in controlled, simplified and fully addressable artificial environments, outside the confines of the living cell.

## Concluding remarks

The bottom-up construction of artificial cells is a burgeoning field, and the advancement and incorporation of microfluidic technologies has played a crucial role in its development. Microfluidics allows membrane-encapsulated artificial cells with cellular dimensions, with a uniform size distribution, with a controlled biomolecular membrane composition and with defined internal content and architecture to be generated rapidly. This is a young field however, and there are several challenges yet to be overcome for it to fulfil its potential in academia, industry and the clinic. Most studies to date have involved low numbers of cells, and have been of a proof of concept nature, with the high-throughput aspect of microfluidics not fully leveraged. Issues surrounding the stability of vesicles, their ability to withstand chemical and mechanical perturbations, the robustness and lifetime of encapsulated biochemical processes and the reproducibility of the microfluidic chips are yet to be adequately tackled. Further miniaturization to micron- and sub-micron regimes is needed for vesicle-based artificial cells to serve functions as drug-delivery vehicles. The high costs associated with cell-free protein expression systems, and with DNA synthesis also present a challenge for scale-up, but one that is being steadily overcome by the biochemical community.

On a broader level, fundamental research on how complex biological systems function and how individual components interact with one another is needed to engineer smarter, more efficient systems. Finally, more effective synergies with the disciplines of top-down synthetic biology, chemical biology, membrane biophysics and macromolecular chemistry will aid further developments. The democratization both of microfluidics (with the emergence of cheap rapid prototyping technologies such as 3D printers) and of cell-free biology (with easy-to-use off-the-shelf kits) will likely help in this regard.

In conclusion, the basic microfluidic platforms for the construction of artificial cells can now be considered to be in place. Microfluidics has also opened up new avenues of research in the discipline, including the generation of multicellular tissue-like structures and for microfabricated devices themselves to serve as an artificial cell chassis. It is expected to have an ever-increasing importance as a platform technology for the construction of artificial cells and one on which the discipline as a whole will become reliant.

## Abbreviations

DIB: droplet interface bilayer
IVTT: *in vitro* transcription and translation
